# Cycle of Disadvantages: An Exploration of Young and Older Female Dementia Caregivers’ Experiences

**DOI:** 10.1093/geroni/igaa057.1128

**Published:** 2020-12-16

**Authors:** Kafayat Mahmoud, Candidus Nwakasi

**Affiliations:** 1 University of Kansas, Lawrence, Kansas, United States; 2 University of Southern Indiana, Evansville, Indiana, United States

## Abstract

Families play an essential role in providing care for older persons in Nigeria, a responsibility that is culturally assigned to women. Caregiving is made even more difficult given the increasing rates of older persons with dementia and the limited capacity of caregivers to provide adequate care. We explored the experiences of two female dementia caregivers and how they cope with other competing responsibilities. The research method employed a case study approach, which followed the lived experiences of two Nigerian women – a 75-year-old woman (Ada) who cares for her husband, and a 35-year old woman (Chika) who cared for her father. The research adopted the life course concept of ‘linked lives’ and the Family Stress Process model to explore and identify the continued embeddedness of persons within their family networks and relationships, which may have deleterious effects on the wellbeing of caregivers. This study revealed that married women providing care to older parents are exposed to domestic violence from their spouses who deem them uncommitted to their (husbands’) families. These women continue to remain in such abusive relationships due to financial dependence and unwillingness to leave their children. Older women caregivers, on the other hand, over time, begin to develop physical limitations, which limit their ability to provide care for their spouses and subsequently must depend on unemployed children who live close by. This study highlights the complicated cycle of exposure to socio-economic and health disadvantages which women experience as caregivers across two generations.

